# The Agnostic Role of Site of Metastasis in Predicting Outcomes in Cancer Patients Treated with Immunotherapy

**DOI:** 10.3390/vaccines8020203

**Published:** 2020-04-28

**Authors:** Andrea Botticelli, Alessio Cirillo, Simone Scagnoli, Bruna Cerbelli, Lidia Strigari, Alessio Cortellini, Laura Pizzuti, Patrizia Vici, Federica De Galitiis, Francesca Romana Di Pietro, Edoardo Cerbelli, Michele Ghidini, Giulia D’Amati, Carlo Della Rocca, Silvia Mezi, Alain Gelibter, Raffaele Giusti, Enrico Cortesi, Paolo Antonio Ascierto, Marianna Nuti, Paolo Marchetti

**Affiliations:** 1 Department of Clinical and molecular oncology, University of Rome “Sapienza”, 00185 Rome, Italy; andrea.botticelli@uniroma1.it (A.B.); paolo.marchetti@uniroma1.it (P.M.); 2 Department of Radiological, Oncological and anatomo-pathological Science, University of Rome “Sapienza”, 00185 Rome, Italy; alessio.cirillo@uniroma1.it (A.C.); bruna.cerbelli@uniroma1.it (B.C.); edoardo.cerbelli91@gmail.com (E.C.); giulia.damati@uniroma1.it (G.D.); silvia.mezi@uniroma1.it (S.M.); agelibter@yahoo.it (A.G.); Enrico.Cortesi@uniroma1.it (E.C.); 3 Department of Medical and Surgical Sciences and Translational Medicine, University of Rome “Sapienza”, 00185 Rome, Italy; 4 Medical Physics Unit, “S. Orsola-Malpighi” Hospital, 40138 Bologna, Italy; lidia.strigari@aosp.bo.it; 5 Department of Biotechnology and Applied Clinical Sciences, University of L’Aquila, 671000 L’Aquila, Italy; alessiocortellini@gmail.com (A.C.); pizzuti8@hotmail.com (L.P.); 6 Division of Medical Oncology 2, IRCCS Regina Elena National Cancer Institute, 00144 Rome, Italy; Patrizia.vici@ifo.gov.it; 7 Istituto Dermopatico dell’Immacolata, 00167 Rome, Italy; degalitiis@yahoo.it (F.D.G.); franci27@live.it (F.R.D.P.); 8 Fondazione IRCCS Ca’ Granda Ospedale Maggiore Policlinico, 20122 Milano, Italy; micheleghidini@outlook.com; 9 Department of Medico-Surgical Sciences and Biotechnology, Polo Pontino, Sapienza University, 00185 Roma, Italy; carlo.dellarocca@uniroma1.it; 10 Sant’Andrea Hospital, Department of clinical and molecular medicine, Sapienza, University of Rome, 00153 Roma, Italy; raffaelegiusti@yahoo.it; 11 Unit of Melanoma, Cancer Immunotherapy and Development Therapeutics, Istituto Nazionale Tumori IRCCS Fondazione G. Pascale, Via Mariano Semmola snc, 80131 Naples, Italy; paolo.ascierto@gmail.com; 12 Department of Experimental Medicine, University Sapienza, 00185 Rome, Italy; marianna.nuti@uniroma1.it

**Keywords:** immunotherapy, agnostic biomarkers, predictive factors, prognostic factors, metastatic sites, melanoma, NSCLC, RCC

## Abstract

Immune checkpoint inhibitors have revolutionized treatment and outcome of melanoma and many other solid malignancies including non-small cell lung cancer (NSCLC) and renal cell carcinoma (RCC). Unfortunately, only a minority of patients have a long-term benefit, while the remaining demonstrate primary or acquired resistance. Recently, it has been demonstrated that the prevalence of programmed death-ligand 1 (PD-L1) and tumor-infiltrating lymphocytes (TILs) varies based on the anatomical site of metastases. In particular, liver seems to have more immunosuppressive microenvironment while both the presence of lymph nodal disease and lung metastases seem to have the highest prevalence of PD-L1 and TILs. The aim of the present study is to investigate the possible role of site of metastases as a predictive factor for response or resistance to immunotherapy in several types of cancer. In this multicenter retrospective study, we enrolled patients with metastatic NSCLC, melanoma, RCC, urothelial, merkel carcinoma, and colon cancer who received immunotherapy from April 2015 to August 2019. Major clinicopathological parameters were retrieved and correlated with patients’ survival outcomes in order to assess their prognostic value and build a useful tool to assist in the decision-making process. A total of 291 patients were included in this study. One hundred eighty-seven (64%) patients were male and 104 (36%) female. The tumor histology was squamous NSCLC in 56 (19%) patients, non-squamous NSCLC in 99 (34%) patients, melanoma in 101 (35%) patients, RCC in 28 (10%) patients, and other tumors in the remaining 7 (2%) patients. The number of metastatic sites was 1 in 103 patients (35%), 2 in 104 patients (36%) and 3 in 84 patients (29%). Out of 183 valuable patients, the entity of response was complete response (CR), partial response (PR), stable disease (SD), and progression disease (PD) in 15, 53, 31, and 79 patients, respectively. Using an univariate analysis (UVA), tumor burden (*p* = 0.0004), the presence of liver (*p* = 0.0009), bone (*p* = 0.0016), brain metastases (*p* < 0.0001), the other metastatic sites (*p* = 0.0375), the number of metastatic sites (*p* = 0.0039), the histology (*p* = 0.0034), the upfront use of immunotherapy (*p* = 0.0032), and Eastern Cooperative Oncology Group (ECOG) Perfomance status (PS) ≥ 1 (*p* < 0.0001) were significantly associated with poor overall survival (OS). Using a multivariate analysis (MVA) the presence of liver (*p* = 0.0105) and brain (*p* = 0.0026) metastases, the NSCLC diagnosis (*p* < 0.0001) and the ECOG PS (*p* < 0.0001) resulted as significant prognostic factors of survival. Regarding the progression free survival (PFS), using a UVA of the tumor burden (*p* = 0.0004), bone (*p* = 0.0098) and brain (*p* = 0.0038) metastases, the presence of other metastatic sites (*p* = 0.0063), the number of metastatic sites (*p* = 0.0007), the histology (*p* = 0.0007), the use of immunotherapy as first line (*p* = 0.0031), and the ECOG PS ≥ 1 (*p* ≤ 0.0001) were associated with a lower PFS rate. Using an MVA, the presence of brain (*p* = 0.0088) and liver metastases (*p* = 0.024) and the ECOG PS (*p* < 0.0001) resulted as predictors of poor PFS. Our study suggests that the site of metastases could have a role as prognostic and predictive factor in patients treated with immunotherapy. Indeed, regardless of the histology, the presence of liver and brain metastases was associated with a shorter PFS and OS, but these results must be confirmed in further studies. In this context, a deep characterization of microenvironment could be crucial to prepare patients through novel strategies with combination or sequential immunotherapy in order to improve treatment response.

## 1. Introduction

The advent of immunotherapy represents a revolutionary event for the treatment of cancer especially for melanoma, advanced non-small-cell lung cancer (NSCLC), renal cell carcinoma (RCC), breast cancer, head and neck, and urothelial cancer both in metastatic and adjuvant settings.

The immune checkpoint inhibitors (ICIs) currently used in clinical practise include the antibody anti-cytotoxic T lymphocyte antigen-4 (CTLA-4 inhibitor) ipilimumab, anti-programmed death 1 (anti PD-1) (nivolumab and pembrolizumab) and anti-programmed death-ligand 1 (anti PD-L1) (atezolizumab, avelumab, and durvalumab) were used as monotherapies as well as in combination with other anticancer drugs [1,2,3,4,5].

These agents have a favorable toxicity profile than chemotherapy or targeted therapies offering the promise of durable clinical benefit, albeit only for a minority of patients [6,7,8,9,10].

Today, the real goal is the selection of the ideal patient who can receive immunotherapy through the identification of specific biomarkers. In particular, programmed death-ligand 1 (PD-L1) expression has been studied in lung cancer, bladder cancer, RCC and breast cancer in order to define its putative prognostic and predictive value even though several limitations have been highlighted [11,12,13,14]. Indeed, to date the PD-L1 expression is available in clinical practice only for the choice of treatment of NSCLC [15,16,17,18,19,20] and breast cancer patients [21,22,23,24].

Furthermore, in lung cancer, tumor mutational burden, on tumor tissue and on circulating tumor DNA (ctDNA), has been investigated [25,26], as a potential biomarker for responsiveness/resistance to immunotherapy. In breast cancer, the levels of tumor-infiltrating lymphocytes (TILs) may also be considered as prognostic factors [27,28,29,30,31]. 

In the context of novel biomarkers, indoleamine 2,3-dioxygenase (IDO) [32,33], the enzymes family involved in tryptophan catabolism, CD73, an immunosuppressive ecto-enzyme involved in the production of adenosine [34,35,36,37,38], and microbiome [39,40,41] seem to have a promising role. The complete and comprehensive immune profile requires simultaneous and dynamic evaluation of many biomarkers that cooperate for the success or failure of the immune response, rather than research on a single biomarker.

Some clinical features are under evaluation to predict the response to immunotherapy [42]. In particular, liver metastases are considered a predictor of worse prognosis in lung cancer, melanoma, and gastrointestinal cancer patients [43] such as the pleuric effusion and brain metastases.

In this scenario, we have already performed a nomogram to predict response to nivolumab in a lung cancer cohort of patients [44]. 

The aim of the present study is to investigate the agnostic role of the metastasis site as a predictive response or resistance factor to immunotherapy in several types of cancer. 

## 2. Materials and Methods

### 2.1. Patients, Treatment, and Outcomes

From April 2015 to August 2019, patients with metastatic NSCLC, melanoma, RCC, urothelial, Merkel carcinoma, and colon-cancer receiving immunotherapy from five different hospitals were enrolled into this retrospective study. Patients were clinically staged with contrast enhanced computed tomography (CT) scan and, if clinically indicated, magnetic resonance imaging (MRI).

The final version of the protocol was approved by the Institutional Ethics Committee (Ethical Committee no. 4421, “Sapienza University”).

Nivolumab, pembrolizumab, atezolizumab, and avelumab were administered intravenously according to approved schedule until disease progression or development of unacceptable toxicity. Tumor response was assessed every 8–12 weeks using immune-related Response Evaluation Criteria in Solid Tumors (i-RECIST) and classified as complete response (RC), partial response (RP), stable disease (SD), and progressive disease (PD). Toxicities were recorded at day 1 of every cycle and classified according to the National Cancer Institute Common Terminology Criteria for Adverse Events (version 4.0). Baseline patients’ clinical condition has been defined with Eastern Cooperative Oncology Group (ECOG) Performance Status (PS).

Progression-free survival (PFS) was defined as the time from patient’s first administration of ICIs until the first progression or in-treatment death. The OS was defined as the time from patient registration to death from any cause. Tumor burden was defined as ‘low’ (≤2 metastatic sites) or ‘high’ (>2 metastatic sites). Patients who did not have an event during the observation time (PD or death) are described as censored.

### 2.2. Statistical Analysis

In the descriptive analysis, quantitative variables were described as mean and range, while qualitative variables as number and percentage and they were analyzed with Fisher’s exact test or Pearson’s Chi-square test. The association of clinic-pathological characteristics and OS and PFS was analyzed by both the univariate and multivariate analyses (UVA and MVA, respectively). PFS and OS were estimated using the Kaplan–Meier method, prognostic clinic-pathological variables deemed of potential relevance with the UVA (assuming a cutoff of *p* < 0.10) were included in the MVA analysis. Discrimination of nomogram was tested by Kaplan–Meier curves and boxplots. A *p* < 0.05 was considered statistically significant. Statistical analyses were performed using R-package software.

## 3. Results

Two hundred ninety-one metastatic patients treated with ICIs were enrolled in this study. The baseline clinical and pathological characteristics, including gender, ECOG PS, histology, primary sites, previous treatment, number, and sites of metastases are reported in Table 1.

One hundred eighty-seven (64%) patients were male and 104 (36%) female. The tumor histology was squamous NSCLC in 56 (19%), non-squamous NSCLC in 99 (34%), melanoma in 101 (35%), clear cell RCC in 28 (10%) and other tumors in the remaining 7 (2%) patients. Baseline ECOG PS, evaluated at the beginning of immunotherapy, was 0, 1, and 2 in 146 (50%), 102 (35%%), 42 (15%) patients, respectively at the baseline imaging evaluation with CT scan, the number of metastatic sites was 1 in 103 patients (35%), 2 in 104 patients (36%), and 3 in 84 patients (29%) as expected based on the natural history of each tumor type. The most frequent metastatic site was lung, detected in 185 patients (64%), and followed by lymph nodes (48%). Bone, liver and brain metastases were detected in 75 (26%), 59 (20%), and 42 (14%) of patients respectively. Data related to immunotherapy are shown in Table 2. In most cases the immunotherapy was planned as a first line treatment (51%). Overall, 94% of patients received immunotherapy as first, second, or third line and the remaining 6% received immunotherapy beyond the fourth line. The most frequently used ICIs were nivolumab (75%) and pembrolizumab (21%), while the anti PD-L1 atezolizumab and avelumab were used in 11 and 1 patients respectively. One hundred and eighty-three out of 291 patients were evaluable for best response to ICIs. Fifteen CR (8%), 53 PR (29%), and 31 SD (17%) were achieved. Overall, disease progression occurred in 79 patients (43%) (Table 2). No high-grade toxicities were reported.

A possible association between disease progression, clinical and pathological characteristics and previous treatment is shown in the Table 3.

**Figure 1:**
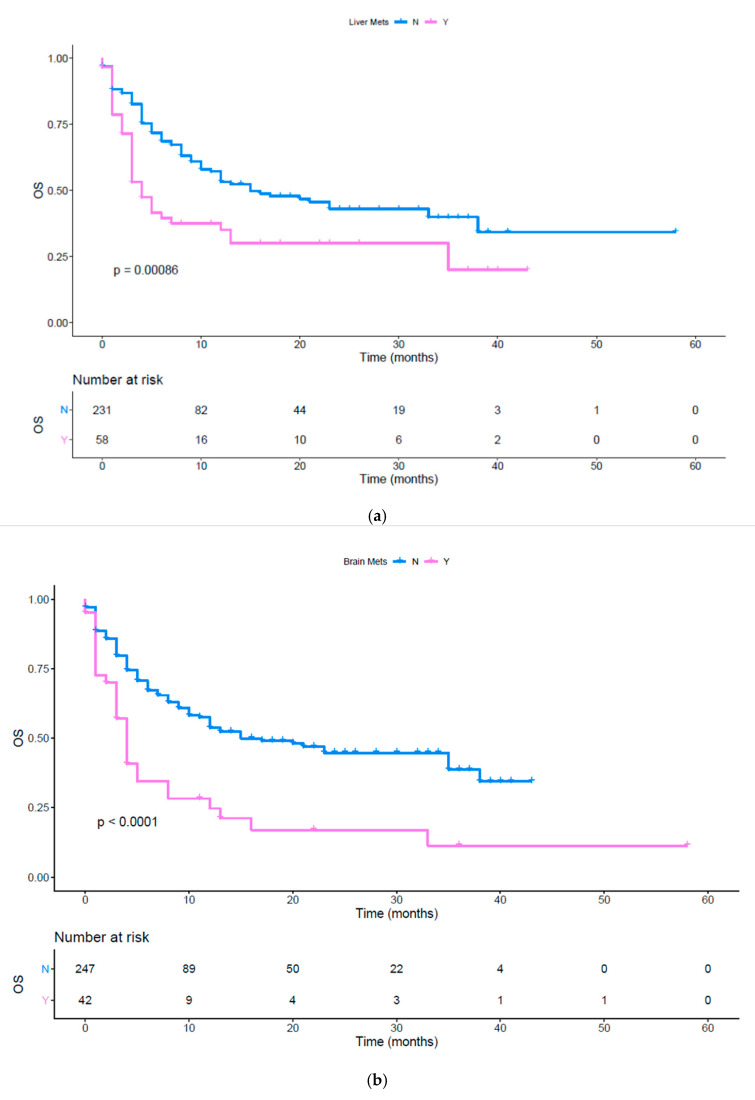

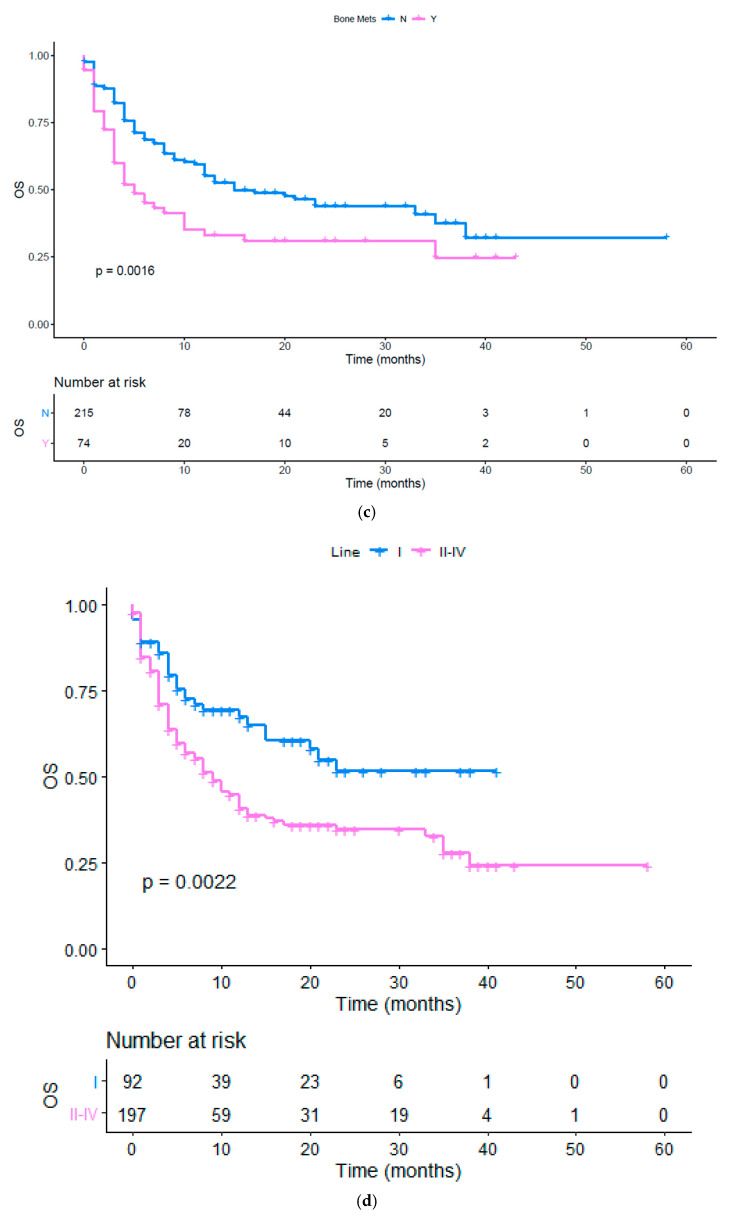

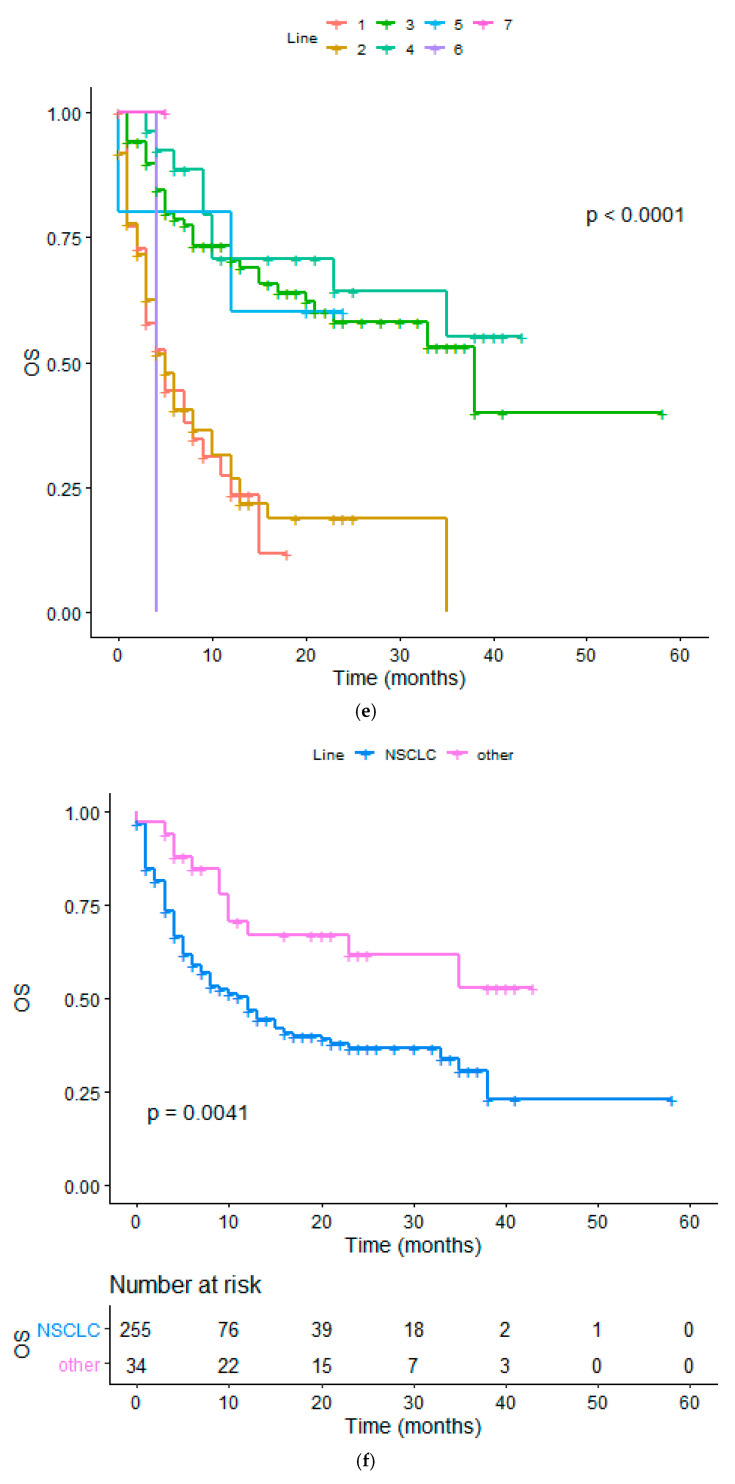
(**a**) OS in patients with and without liver metastases; (**b**) OS in patients with and without brain metastases; (**c**) OS in patients with and without bone metastases; (**d**) OS in patients treated with immunotherapy upfront or in subsequent lines; (**e**) OS in different histologies; (**f**) OS in patients affected by squamous and non-squamous NSCLC or other cancers.

Performing a univariate analysis, tumor burden (*p* = 0.0004), the presence of liver (Figure 1a, *p* = 0.0009), bone (Figure 1b, *p* = 0.0016), brain metastases (Figure 1c, *p* < 0.0001), the other metastatic sites (*p* = 0.0375), the number of metastatic sites (Figure S1, *p* = 0.0039) and ECOG PS ≥ 1 (Figures S2 and S3, *p* < 0.0001) were significantly associated with lower OS (Table 3). Moreover, we evaluated the impact of immunotherapy in first line versus second or subsequent lines. Patients treated upfront with immunotherapy had a better OS compared to a later administration (Figure 1d, *p* = 0.0022). Furthermore, we evaluated OS between different cancer histologies, highlighting a worse prognosis in patients affected by both squamous and non-squamous NSCLC compared to other cancers (Figure 1e,f, *p* < 0.001 and *p* = 0.0044 respectively). The 24-month OS fraction evaluated for several patients’ characteristics is reported in Table 4. Using a MVA of the use of upfront ICIs did not result as independent predictor. The metastatic site of liver (*p* = 0.0051) and brain (*p* = 0.0021), the NSCLC histology (both squamous and non-squamous, *p* < 0.0001) and the ECOG PS (*p* < 0.0001) were confirmed as prognostic factors at the multivariate analysis (Table 5).

Figure 1a shows the OS difference in patients with and without liver metastases pink line: patients with liver metastases; blue line: patients without liver metastases; OS: overall survival; Tick marks indicate censored data.

Figure 1b shows OS difference in patients with and without brain metastases (*p* < 0.0001) pink line: patients with brain metastases; blue line: patients without brain metastases; OS: overall survival. Tick marks indicate censored data.

Figure 1c shows the OS difference in patients with and without bone metastases (*p* < 0.0016) pink line: patients with bone metastases; blue line: patients without bone metastases; OS: overall survival. Tick marks indicate censored data.

Figure 1d shows the OS difference in patients treated with immunotherapy in fist line vs. second or subsequent lines (*p* < 0.0022) pink line: first line; blue line: subsequent lines; OS: overall survival. Tick marks indicate censored data.

Figure 1e shows the OS in patients affected by different cancers (*p* < 0.0001) red line (1): NSCLC squamous; yellow line (2): NSCLC non squamous; light green line (3): melanoma; dark green line (4): RCC; light blue line (5): Urothelial cancer; violet line (6): merkel cell; pink line (7): colon; OS: overall survival; NSCLC: non-small cell lung cancer; RCC: renal cell carcinoma Tick marks indicate censored data.

Figure 1f shows the OS in patients affected by both squamous and non-squamous NSCLC versus patients affected by other cancer histologies. blue line: NSCLC squamous and non-squamous; pink line: other histologies; OS: overall survival; NSCLC: non-small cell lung cancer; Tick marks indicate censored data.

**Figure 2:**
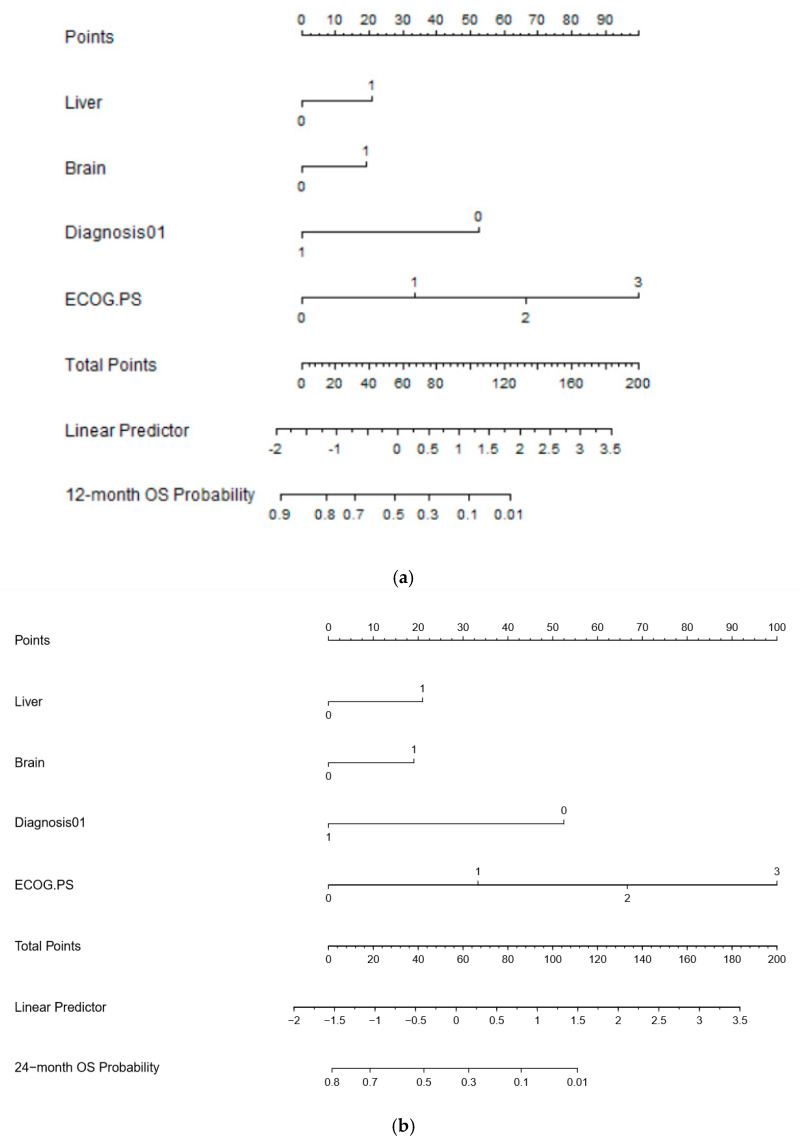

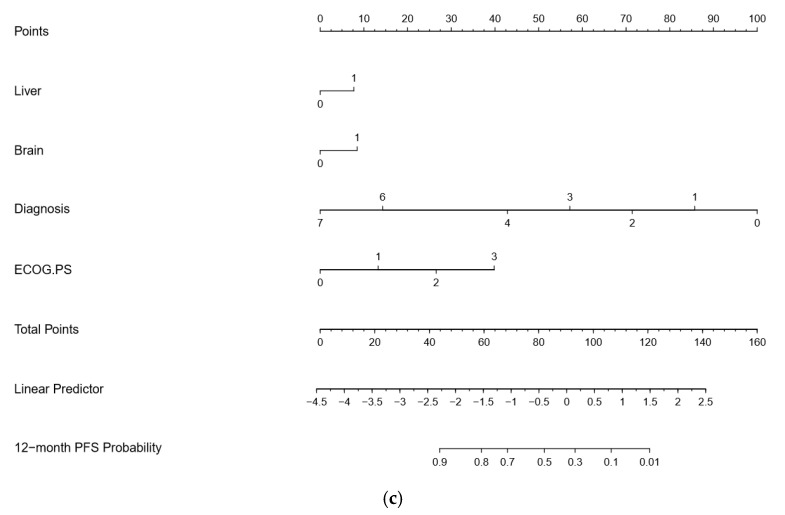
(**a**) 12-month OS nomogram; (**b**) 24-month OS nomogram; (**c**) 12-month PFS nomogram.

**Figure 3:**
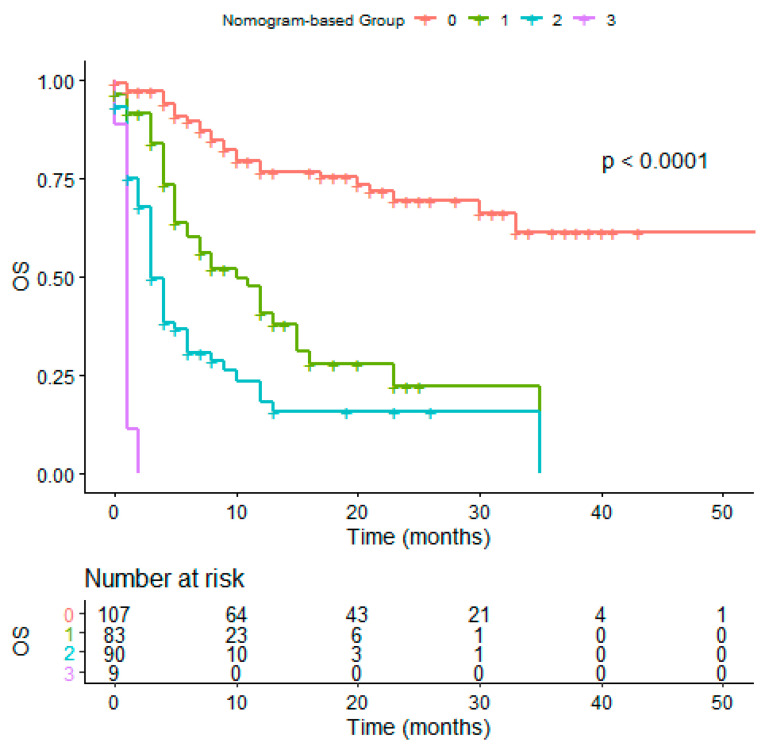
OS in nomogram-based groups.

Based on the estimated regression coefficients in the Cox analysis, a prognostic nomogram including the presence of liver and brain metastases, ECOG PS, and histology was developed to assign survival probability at 12 and 24 months (Figure 2a,b, respectively) in patients treated with immunotherapy. The prognostic nomogram was developed including parameters which were significant to multivariate analysis to assign survival probability at 12 and 24 months after immunotherapy treatment commencement. Kaplan-Meier curves, according to the range of total points, highlighted the appropriateness of distinguish the patients’ survival in all the subgroups. Based on the result obtained with the nomogram on the studied population, we divided the patients into quartiles (group from 0 to 3). We highlighted a significant difference in OS between groups, confirming the predictive efficacy of the nomogram (Figure 3, *p* < 0.001).

Figure 2a,b: To use the nomogram, a vertical line needs to be delineated to the point raw to assign point values for each variable. Thereafter, the corresponding points are to be summed to obtain the total points. Finally, from the total points a vertical line needs to be drawn to get the value of 24-month OS probability. The presence of liver and brain metastases corresponds to 21 and 20 points, respectively, while the ECOG PS of 0 corresponds to 0 points. A diagnosis of cancer different from NSCLC correspond to 0 points. The C-indexes for OS models was 0.797 and calibration of the nomogram for OS was considered adequate (Figures S3 and S4). Figure 2a,b footnotes: Eastern Cooperative Oncology 

To use the nomogram, a vertical line needs to be delineated to the point raw to assign point values for each variable. Thereafter, the corresponding points are to be summed to obtain the total points. Finally, from the total points, a vertical line needs to be drawn to get the value of 12-month PFS probability. Histologies have a larger impact on PFS, taking into account the different natural history and timing of immunotherapy treatment in different cancers. For this reason, cancer histology has been specified in this PFS nomogram. The C-indices for PFS models were 0.665 and the calibration of the nomogram for PFS was considered adequate.

Diagnosis: 0—squamous NSCLC; 1—non-squamous NSCLC; 2—melanoma; 3—RCC; 4—urothelial cancer; 6—Merkel; 7—other histologies. Figure 2b footnotes: NSCLC: non-small cell lung cancer; RCC: renal cell carcinoma; Eastern Cooperative Oncology Group (ECOG) performance status (PS); PFS: progression free survival.

Figure 3 shows the OS difference in patients divived in quartile using the OS nomogram result (*p* < 0.0001). Red line: patients in the fourth quartile (75 to 100); green line: patients in the third quartile; blue line: patients in the second quartile; purple line: patients in the first quartile using the OS nomogram; OS: overall survival. Tick marks indicate censored data.

**Figure 4:**
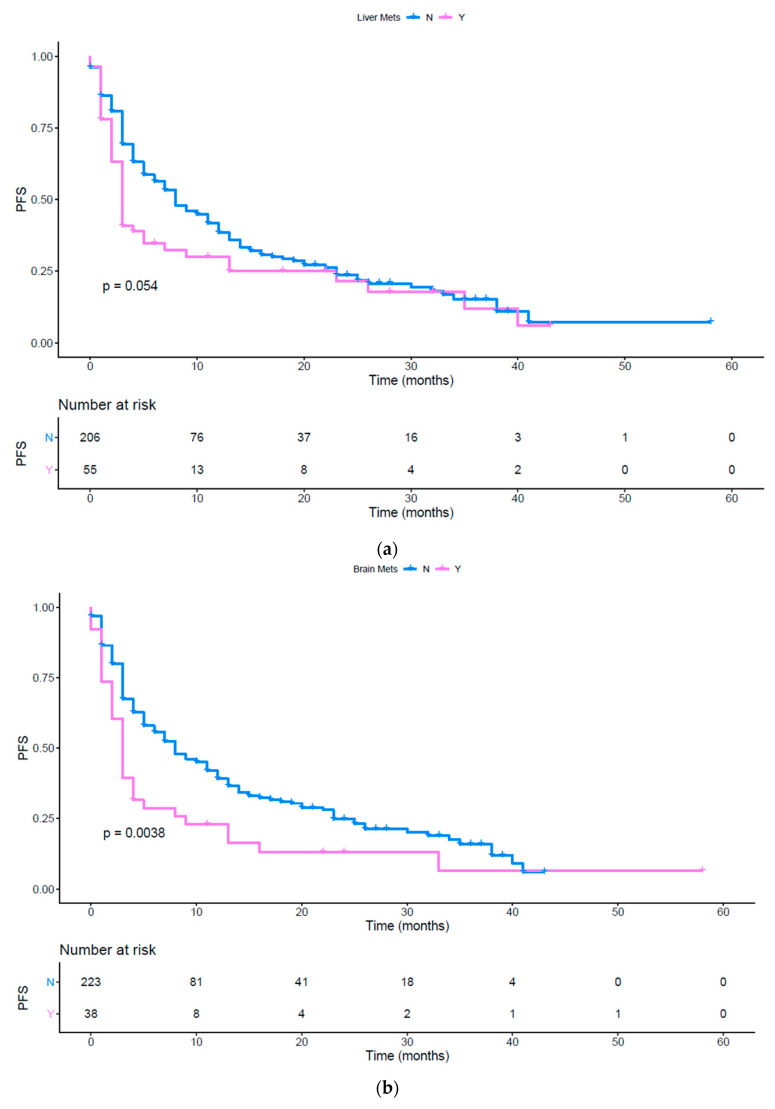

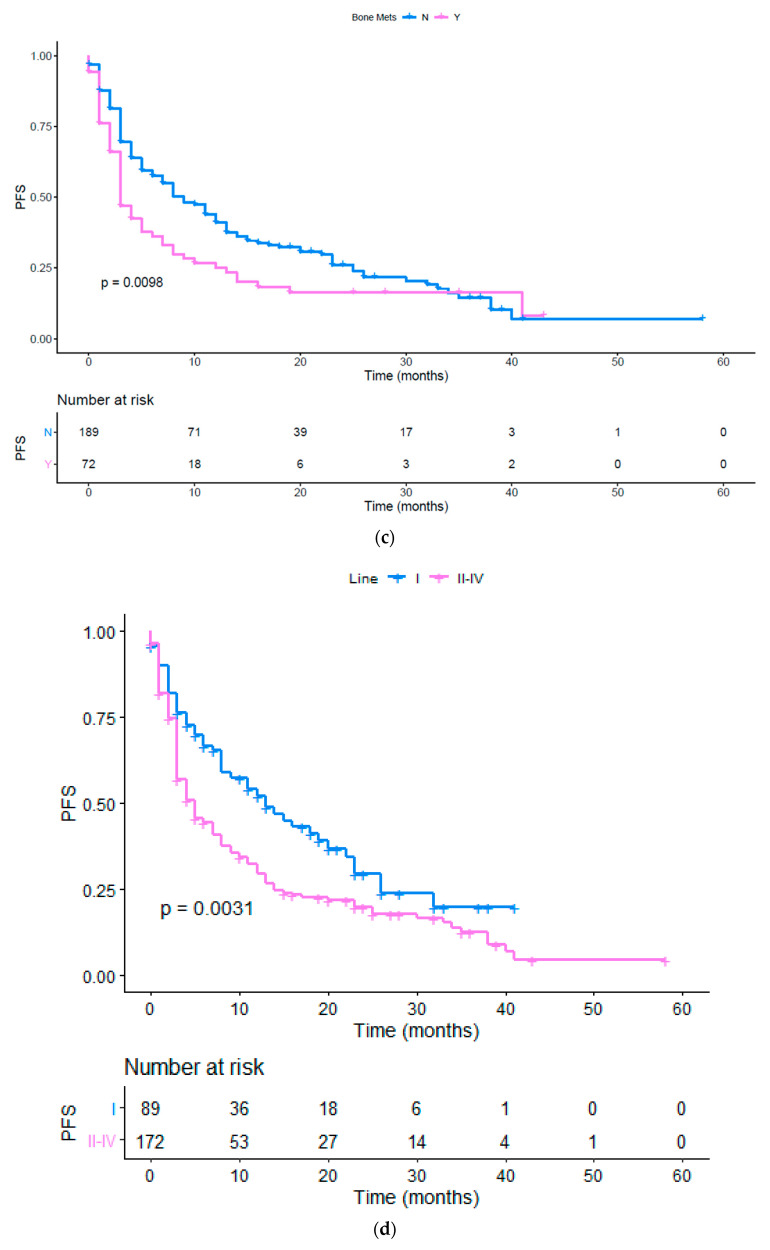

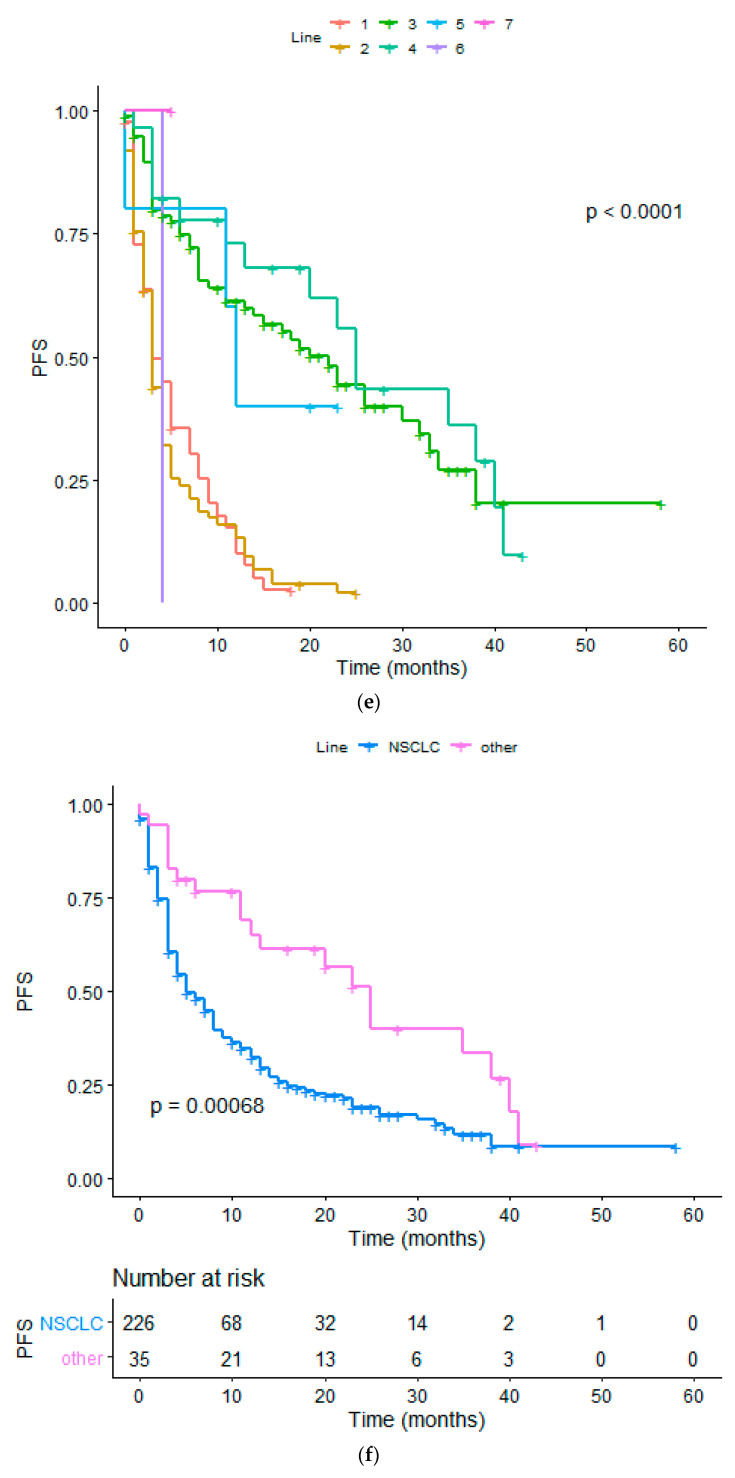
(**a**) PFS in patients with and without liver metastases; (**b**) PFS in patients with and without brain metastases; (**c**) PFS in patients with and without bone metastases; (**d**) PFS in patients treated with immunotherapy upfront or in subsequent lines; (**e**) PFS in different histologies; (**f**) PFS in patients affected by squamous and non-squamous NSCLC or other cancers.

Using a UVA of the tumor load (*p* = 0.0011), the presence of bone (Figure 4c, *p* = 0.0098), brain (Figure 4b, *p* = 0.0038) metastases, the presence of other metastatic sites (*p* = 0.0063), the number of metastatic sites (Figure S5, *p* = 0.0007) and the ECOG PS ≥ 1 (Figure S6; *p* = <0.0001) were associated to a poor PFS (Table 2). The presence of liver metastases showed a tendency to be statistically significant and was included in the subsequent MVA (Figure 4a, *p* = 0.0535). Moreover, as previously done for the OS analysis, we performed a UVA to evaluate the impact of upfront or subsequent treatment with immunotherapy. First line treatment resulted in an improved PFS (Figure 4d, *p* < 0.003), but this trend was not confirmed at MVA. Finally, NSCLC patients’ subgroup had a worse PFS compared to the others (Figure 4e,f, *p* < 0.0001 and *p* = 0.0068 respectively). A significant association between an impaired PFS and the presence of brain metastases (*p* = 0.0088), liver metastases (*p* = 0.024) was confirmed with the MVA. Brain and liver metastases were confirmed as independent predictors of poor response to immunotherapy. Moreover, NSCLC diagnosis (*p* = 0.0001) and ECOG PS (*p* < 0.0001) were also strong predictors of poor clinical outcomes (Table 6).

Figure 4a shows the PFS difference in patients with and without liver metastases (*p* = 0.054) pink line: patients with liver metastases; blue line: patients without liver metastases; PFS: Progression Free Survival. Tick marks indicate censored data.

Figure 4b shows the PFS difference in patients with and without brain metastases (*p* = 0.0038) pink line: patients with brain metastases; blue line: patients without brain metastases; PFS: Progression Free Survival. Tick marks indicate censored data.

Figure 4c shows the PFS difference in patients with and without bone metastases (*p* = 0.0098) pink line: patients with bone metastases; blue line: patients without bone metastases; PFS: Progression Free Survival. Tick marks indicate censored data.

Figure 4d shows the PFS in patients treated with immunotherapy upfront or later (*p* = 0.0031) pink line: patients treated with immunotherapy as first line; blue line: patients treated with immunotherapy as second or later lines; PFS: progression free survival. Tick marks indicate censored data.

Figure 4e shows the PFS in patients affected by different cancers (*p* < 0.0001) red line (1): NSCLC squamous; yellow line (2): NSCLC non squamous; light green line (3): melanoma; dark green line (4): RCC; light blue line (5): Urothelial cancer; violet line (6): Merkel cell; pink line (7): colon; PFS: progression free survival; NSCLC: non-small cell lung cancer; RCC: renal cell carcinoma. Tick marks indicate censored data.

Figure 4f shows the PFS in patients affected by both squamous and non-squamous NSCLC versus patients affected by other cancer hystologies (*p* = 0.00068). blue line: NSCLC squamous and non-squamous; pink line: other histologies; PFS: progression free survival; NSCLC: non-small cell lung cancer. Tick marks indicate censored data.

Based on the estimated regression coefficients in the Cox analysis, a prognostic nomogram that included liver and brain metastases, diagnosis, and ECOG PS was developed to assign the PFS probability at 12 months (Figure 2b) after immunotherapy treatment commencement.

## 4. Discussion

Immunotherapy has radically changed the approach to the cancer patient in hard to treat malignancies. Nevertheless, the large phase III studies showed that only a subset of patient’s benefits from immunotherapy, with lasting responses and very limited side effects. Patient’s selection of treatment is becoming a dominant issue to avoid unnecessary progressions of disease and improving the overall outcome. Moreover, the data available to date do not allow us to identify predictive biomarkers of response to the treatment with anti PD-1, anti PD-L1 and anti CTLA 4. Aware of the difficulty of finding a reliable biomarker in the complex and dynamic immune system, research is focusing on what would appear to be a surrogate such as tumor mutational burden or other molecular signatures.

In this regard, some clinical features (ECOG PS, lactate dehydrogenase, etc.) would seem to be able to drive the choice of treatment even if the immunological rationale is not always clear.

In this context, it is also known how the immunological microenvironment of metastatic disease can be different considering the specific organ, with a possible impact on the response to immunotherapy. The site of the metastases, regardless of the primitive cancer, impacts in our study on the response to immunotherapy as well as on the prognosis, suggesting that the site of metastasis could have an agnostic predictive and prognostic role in predicting response to immunotherapy. Our data point as disease progression is related to the specific site of metastases, which influences the site-specific progression. Furthermore, the few responses to treatment in liver and brain metastatic sites have been limited and transient.

Indeed, regardless of the histology, in patients treated with immunotherapy the presence of liver or brain metastases seems to be associated with a shorter PFS and OS, as previously demonstrated in NSCLC [44] and melanoma [45]. The biological rationale has not yet been fully explained but some preliminary data in the literature support the idea of considering the immune system as a specific organ.

To date, the role of metastatic disease in the liver is considered as a negative predictive and prognostic factor related to patients receiving chemotherapy-based treatment, including multimodal and aggressive strategy.

However, in the novel context of immunotherapy and immune biomarkers, the liver site of metastasis appears to play a relevant negative predictive role in tumor response. In particular, it was demonstrated that the poor response to immunotherapy is associated with liver metastasis in melanoma patients treated with ipilimumab and nivolumab [45] and in NSCLC patients treated with nivolumab [43,44]. The peculiar immunological behavior of patients with liver metastases could be related to the immunological context of liver microenvironment that seems to be ‘colder’ than the primary tumor or than another metastatic site such as lung or lymph node. Several mechanisms of immune-escape have been postulated, such as the incomplete activation of CD8+ lymphocytes T (T cells), the trapping and the deletion of activated CD8+ cells, the inefficient activity of CD4+ T cells, and the activation of regulatory T cells by Kupffer cells [45].

Brain metastases occur in 20% of cancer patients, especially in those with lung, breast, melanoma, or renal cell carcinoma, determining a poor prognosis. The role of ICIs in these patients is an open issue due to the poor results. Indeed, the use of ipilimumab as a single agent for melanoma brain metastases resulted in disease control rates for only 10% and 25% of patients treated with or without steroids, respectively. Single agent pembrolizumab was associated with an intracranial response rate in 33% of patients in NSCLC and 22% in melanoma patients [46]. However, the role of brain metastases in patients treated with ICIs can be explained through several mechanisms such as activation of regulatory lymphocytes T (Treg), lymphopenia, reduction of T-cell responses, and deficiency of interleukin 2 (IL-2) signaling that result in an immunosuppressive microenvironment [47].

Despite ICIs having shown significant activity on visceral disease, the efficacy in patients with bone metastases is not well explored. Indeed, the predictive and prognostic role of bone metastases is still controversial and unclear and it could be explained by the role of niches and pathologic bone loss that could hinder immune activation, compromising long-lived memory T and B lymphocytes and the production of cytotoxic T cell [48]. Interestingly, T-regs could be crucial in determining equilibrium between osteoclastic and osteoblastic activity. Indeed, in rheumatic arthritis, it was demonstrated that T-regs are characterized by bone protective effect by direct inhibition of osteoclastogenesis as a negative feedback [49]. The unfavorable outcome in patients with bone metastases could be related to a T-reg-enriched immunosuppressive microenvironment. Our study suggests that bone metastases could be considered both predictive and prognostic factors, but further studies on a larger population are needed.

Of note, applying a UVA to liver, bone, and brain metastases resulted in a significant association with worse PFS and OS compared to other sites, while when using a MVA, only brain and liver metastatic sites were confirmed as independent predictive and prognostic factors.

Indeed, these sites present several peculiar immunological behaviors representing a “bumper site for immunotherapy” characterized by immune suppressive or immune cold microenvironment and suggesting that we need to consider both circulating and organ-specific immunity.

In order to translate these data into clinical practice, we have developed two nomograms for OS and PFS, that are based on easily available and inexpensive clinical factors that have showed a good performance in predicting individual PFS and OS probability among cancer patients treated with immunotherapy. Building a predictive model of response to immunotherapy treatment is currently of primary importance for clinicians and cancer patients. In our study, the site of metastatic spread was able to condition the response to ICIs, PFS, and OS of patients regardless of the primary tumor and its histology. Metastatic cell recognition and killing by lymphocytes seems to be dependent upon the organ specific microenvironment that, if oriented to immune suppression, could result as a specific immune sanctuary. The present study has some limitations to be acknowledged. This is a retrospective cohort study thereby with potential for inherent biases. Still, external prospective validation is required to assess reproducibility and generalizability of our results.

## 5. Conclusions

Specific sites of metastases could have an agnostic role in identifying patients resistant to upfront mono-immunotherapy with anti-PD1/PD-L1 or anti-CTLA-4. In this context, a nomogram to predict outcomes and a deep characterization of the site-specific microenvironment could be crucial to select these patients with poor prognosis through novel strategies with combination or sequential drugs, to overcome resistances and improve immunotherapy results. 

## Figures and Tables

**Table 1:** Baseline characteristics and demographics.

Characteristics	Patients N	(%)
**Gender**		
Male	187	64%
Female	104	36%
**Histology**		
NSCLC ¹ squamous	56	19%
NSCLC ¹ non squamous	99	34%
Melanoma	101	35%
RCC ²	28	10%
Other	7	2%
**Metastatic Sites**		
1	103	35%
2	104	36%
3	84	29%
**Sites of Metastases**		
Lymph node	140	48%
Liver	59	20%
Lung	185	64%
Bone	75	26%
Brain	42	14%
**ECOG PS ³**		
0	146	50%
1	102	35%
2	42	15%

^1^ NSCLC: non-small cell lung cancer; ^2^ RCC: renal cell carcinoma; ^3^ ECOG PS Eastern Cooperative Oncology Group Performance Status.

**Table 2:** Immunotherapy treatments and best response.

Characteristics	Patients N	(%)
**Immunotherapy Regimen**		
Nivolumab	219	75%
Pembrolizumab	60	21%
Atezolizumab	11	4%
Avelumab	1	0%
Durvalumab	0	0%
**Line of Treatment with Immunotherapy**		
1st	94	32%
2nd	147	51%
3rd	33	11%
4th	11	4%
5th or more	4	2%
**Best Response to Immunotherapy**		
PD	79	43%
CR	15	8%
PR	53	29%
NV	5	3%
SD	31	17%
ALL	183	100%

Response Evaluation Criteria in Solid Tumors Criteria (i-RECIST): complete response (RC); partial response (RP); stable disease (SD); progressive disease (PD).

**Table 3:** Univariate analysis for overall survival and progression free survival.

Characteristics	OS		PFS	
	Hazard Ratio (95% CI)	*p*-Value	Hazard Ratio (95% CI)	*p*-Value
**Age ≥ 70**	0.833 (0.568 to 1.191)	0.302	1.044 (0.759 to 1.449)	0.7716
**Gender**	1.140 (0.797 to 1.653)	0.4571	1.174 (0.874 to 1.618)	0.2705
**Tumor burden**	0.545 (0.369 to 0.749)	**0.0004**	0.635 (0.448 to 0.817)	**0.0011**
**Lymphnodes**	1.173 (0.833 to 1.691)	0.3413	1.215 (0.919 to 1.673)	0.1585
**Liver**	0.546 (0.293 to 0.727)	**0.0009**	0.728 (0.458 to 1.005)	0.0535
**Bone**	0.575 (0.334 to 0.774)	**0.0016**	0.679 (0.436 to 0.892)	**0.0098**
**Brain**	0.431 (0.168 to 0.507)	**<0.0001**	0.593 (0.307 to 0.796)	**0.0038**
**Other met sites**	1.476 (1.022 to 2.140)	**0.0375**	1.518 (1.131 to 2.113)	**0.0063**
**N of metastatic sites > 2**	0.818 (0.312 to 1.999)	0.6198	1.303 (0.609 to 2.746)	0.5025
**Diagnosis(NSCLC vs. other)**	2.277 (1.254 to 3.143)	**0.0034**	2.093 (1.320 to 2.822)	**0.0007**
**ECOG PS ≥ 1**	0.281 (0.170 to 0.356)	**<0.0001**	0.446 (0.275 to 0.518)	**<0.0001**
**Line (I vs. II or more)**	0.566 (0.398 to 0.831)	**0.0032**	0.632 (0.459 to 0.854)	**0.0031**

Eastern Cooperative Oncology Group (ECOG) performance status (PS); OS: overall survival; PFS: progression free survival.

**Table 4:** 24-month overall survival (%).

Characteristics	24-M OS Group 1 (%)	24-M OS Group 2 (%)
**Tumor burden** (low vs. high)	49.7	31.5
**Liver met.** (no vs. yes)	42.6	30.5
**Bone met.** (no vs. yes)	43.5	31
**Brain met.** (no vs. yes)	44.5	17
**ECOG PS** (0 vs. ≥1)	60	20.4
**Diagnosis** (other vs. NSCLC)	58.6	17.1
**Line** (1st vs. 2nd or more)	51.4	34.3

Table 4 shows patients 24 months overall survival for different characteristic. Each characteristic is declined as dichotomous variable. Group 1 column indicates 24 months OS for the first modality and Group 2 column for the second modality of each variable (characteristic). 24-month OS is expressed as % of patients alive at 24 months. Table 4 footnotes: Eastern Cooperative Oncology Group (ECOG) performance status (PS); OS: overall survival; PFS: progression free survival; Met.: metastases; NSCLC: non-small cell lung cancer.

**Table 5:** Multivariate analysis for OS

MVA for Overall Survival (*p* < 0.0001)				
Covariate	b	Exp (b)	95% CI of Exp (b)	*p*
Liver	0.5512	1.7353	1.1824 to 2.5467	**0.0051**
Brain	0.4889	1.6306	1.0786 to 2.4650	**0.0211**
NSCLC vs. others	−1.3823	0.251	0.1707 to 0.3691	**<0.0001**
ECOG PS	0.8449	2.3278	1.8642 to 2.9067	**<0.0001**

Table 5 shows that liver and brain metastases are independent predictors of poor OS regardless of histology (*p* = 0.0051 and *p* = 0.0211 respectively). Table 5 footnotes: MVA: multivariate analysis; OS: overall survival: NSCLC: non-small cell lung cancer; Eastern Cooperative Oncology Group (ECOG) performance status (PS). Significant p-value in bold.

**Table 6:** Multivariate analysis for PFS.

MVA for PFS (*p* < 0.0001)				
Covariate	b	Exp (b)	95% CI of Exp (b)	*p*
Liver	0.4003	1.4923	1.0563 to 2.1082	**0.0239**
Brain	0.4151	1.5145	1.0359 to 2.2142	**0.0088**
NSCLC vs. others	−0.6067	0.5451	0.4642 to 0.6402	**<0.0001**
ECOG PS	0.8243	2.2802	1.6958 to 3.0660	**<0.0001**

Table 6 shows that liver and brain metastases are independent predictors of poor PFS regardless of histology (*p* = 0.0243 and *p* = 0.0088 respectively). Table 6 footnotes: MVA: multivariate analysis; PFS: progression free survival; NSCLC: non-small cell lung cancer; Eastern Cooperative Oncology Group (ECOG) performance status (PS). Significant *p*-value in bold.

## Supplementary Materials

The following are available online at https://www.mdpi.com/2076-393X/8/2/203/s1. Figure S1: OS in patients with ≤ or >2 number of metastases; Figure S2: OS in patients with PS 0 vs PS 1-3; Figure S3: OS in PS 0 to 3 subgroups; Figure S4: calibration for 24 months OS nomogram; Figure S5: PFS in patients with ≤2 or >2 number of metastases; Figure S6: OS in PS 0 to 3 subgroups.
