# Supplementary figures and images for: The Agnostic Role of Site of Metastasis in Predicting Outcomes in Cancer Patients Treated with Immunotherapy
Source: Vaccines (Basel). 2020 Apr 28;8(2):203. doi: 10.3390/vaccines8020203 (PMC7349154; doi:10.3390/vaccines8020203)

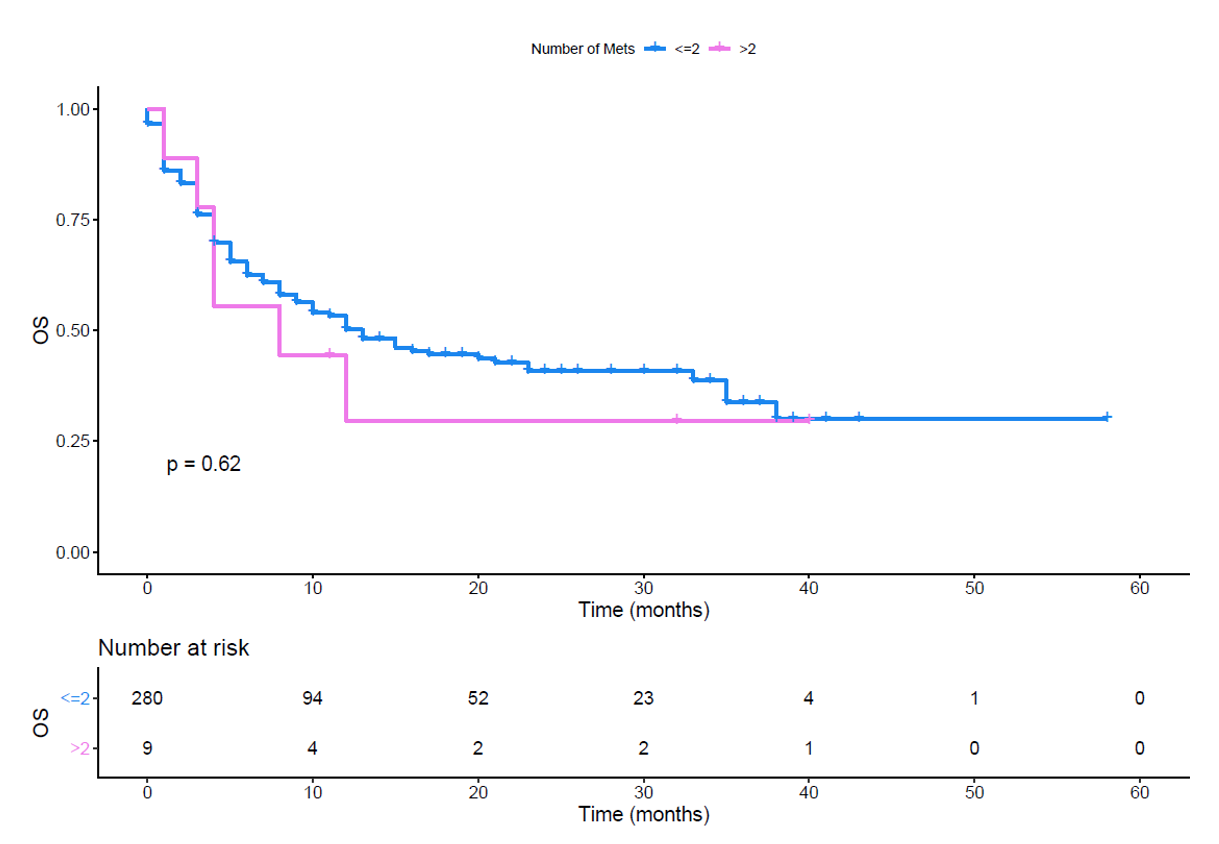

Supplement: Supplementary file 1 [file vaccines-08-00203-s001.zip › suppl 1.tif]

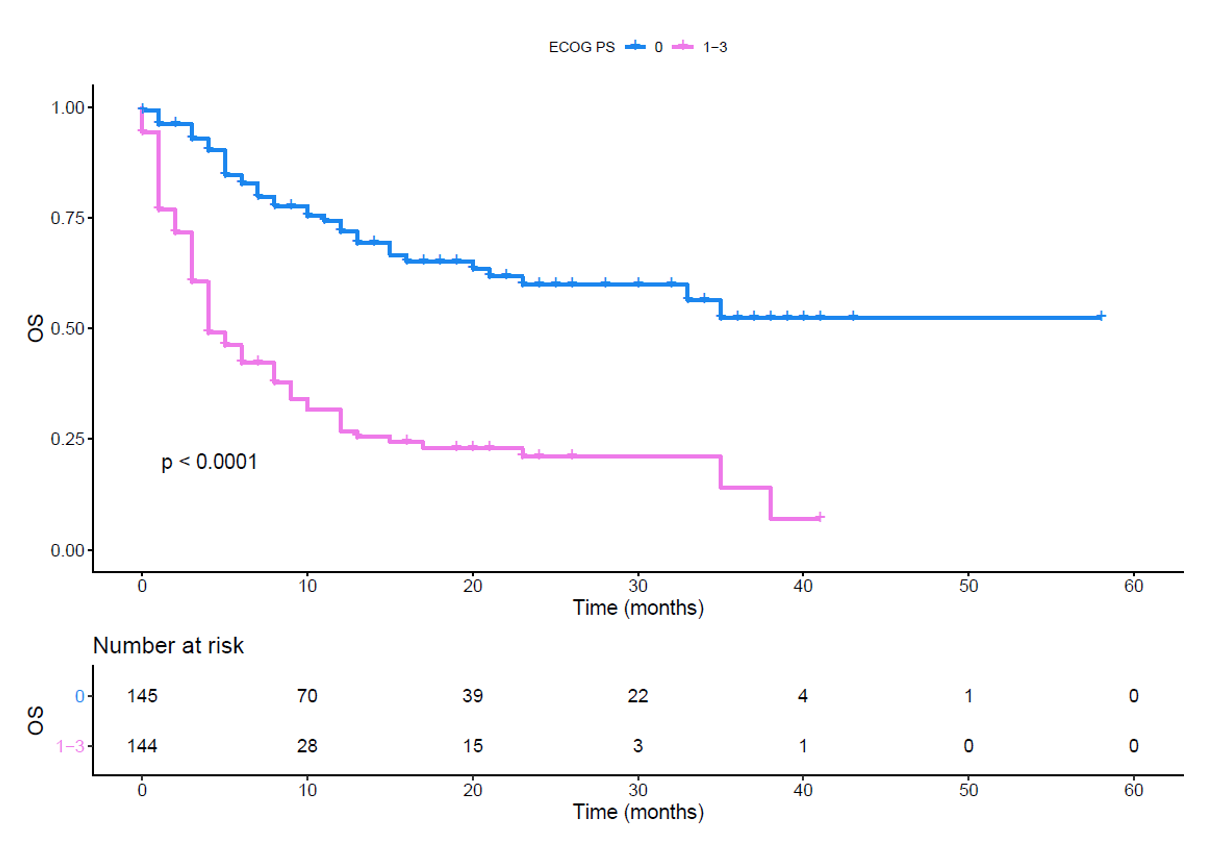

Supplement: Supplementary file 1 [file vaccines-08-00203-s001.zip › suppl 2.tif]

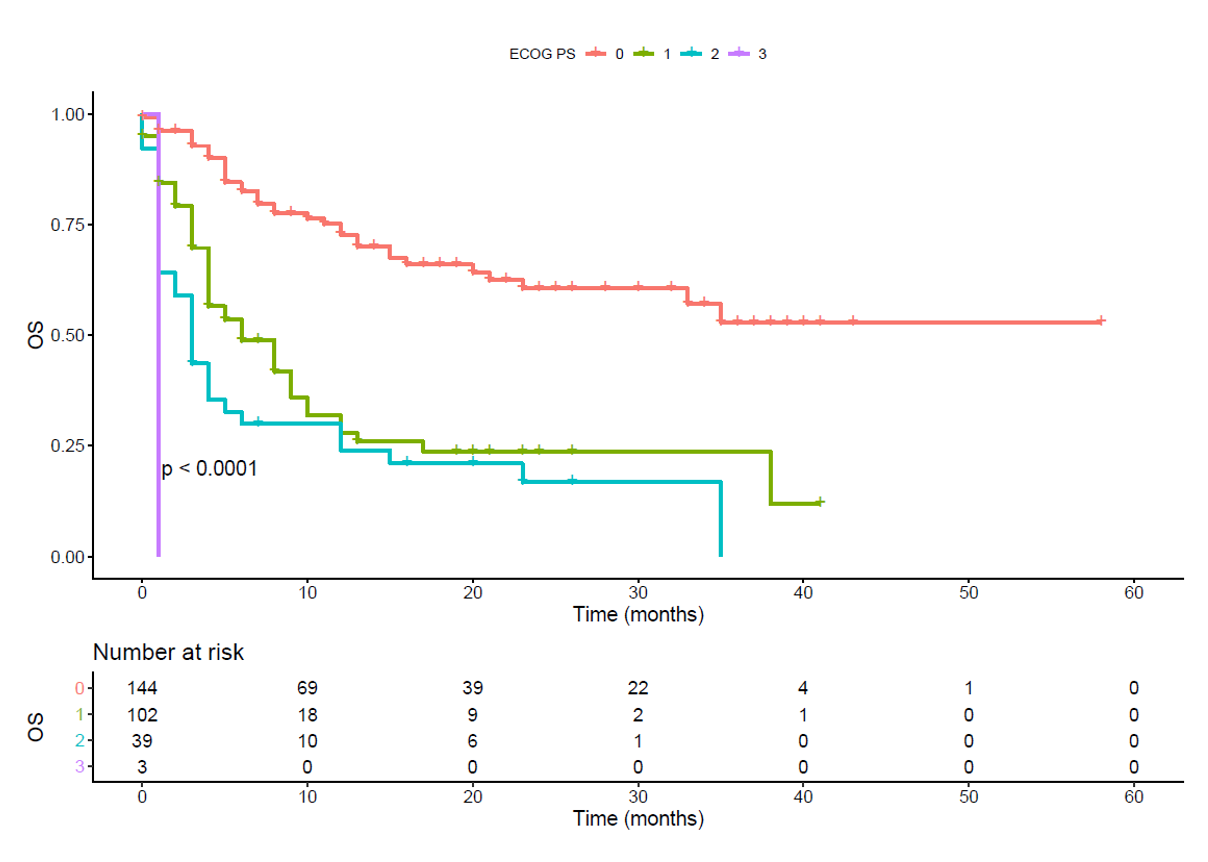

Supplement: Supplementary file 1 [file vaccines-08-00203-s001.zip › Suppl 3.tif]

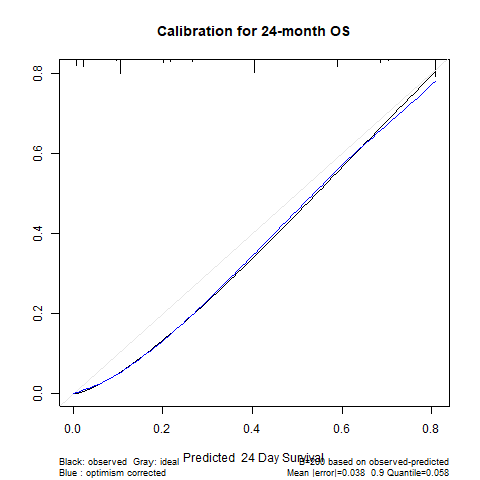

Supplement: Supplementary file 1 [file vaccines-08-00203-s001.zip › suppl 4.tif]

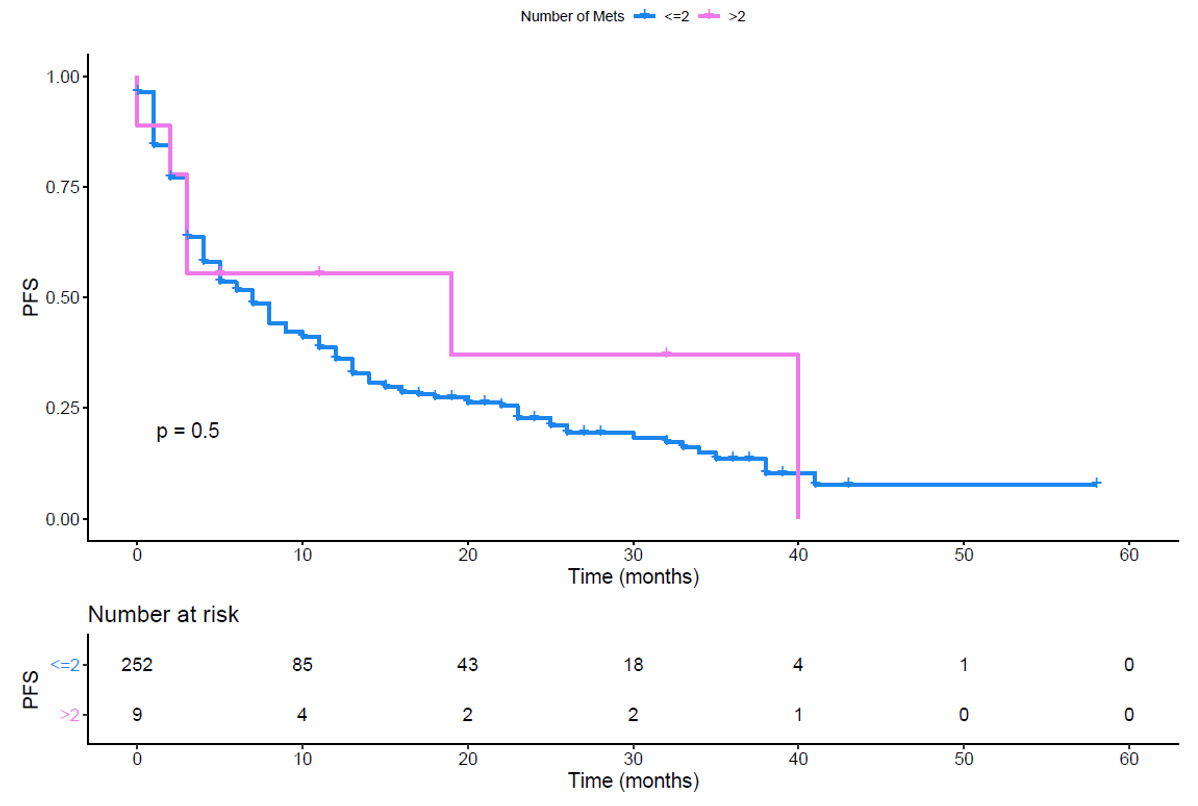

Supplement: Supplementary file 1 [file vaccines-08-00203-s001.zip › suppl 5.tif]

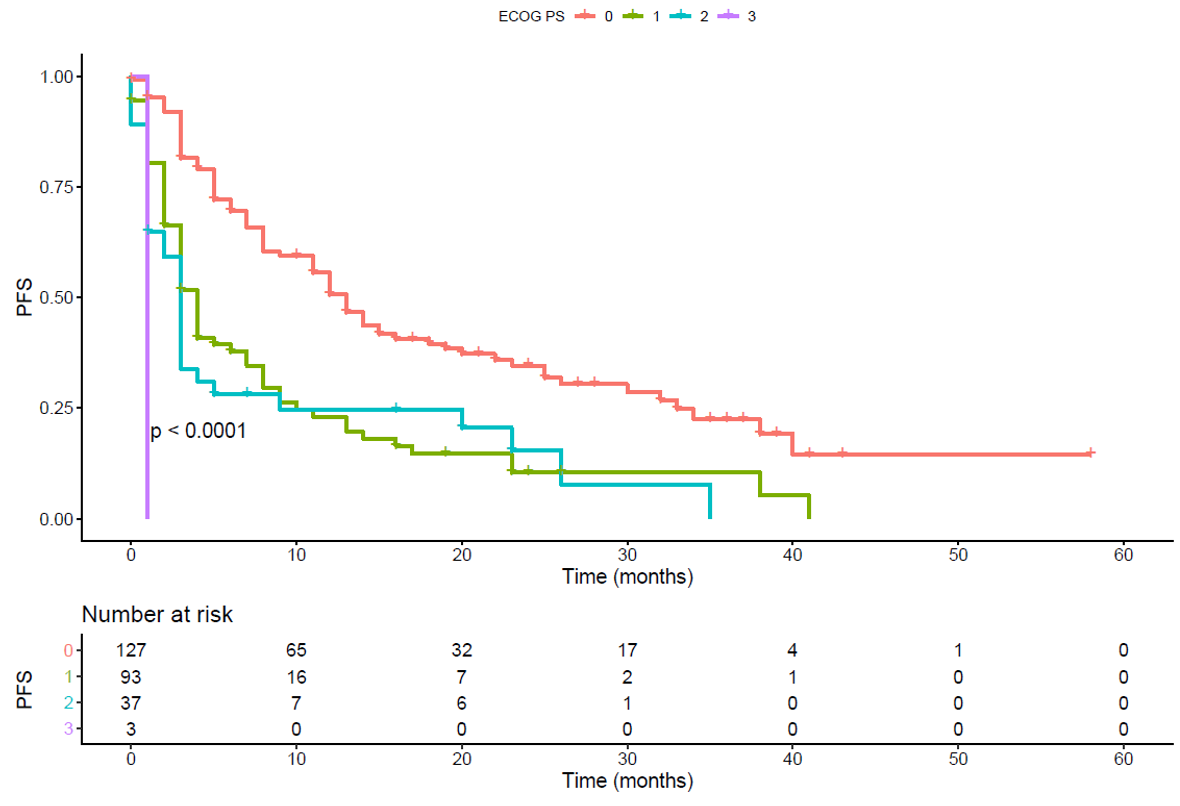

Supplement: Supplementary file 1 [file vaccines-08-00203-s001.zip › suppl 6.tif]
